# Development of a deep learning-based software for calculating cleansing score in small bowel capsule endoscopy

**DOI:** 10.1038/s41598-021-81686-7

**Published:** 2021-02-24

**Authors:** Ji Hyung Nam, Youngbae Hwang, Dong Jun Oh, Junseok Park, Ki Bae Kim, Min Kyu Jung, Yun Jeong Lim

**Affiliations:** 1 Division of Gastroenterology, Department of Internal Medicine, Dongguk University Ilsan Hospital, Dongguk University College of Medicine, Goyang, Republic of Korea; 2 Department of Electronics Engineering, Chungbuk National University, Cheongju, Republic of Korea; 3 Department of Internal Medicine, Digestive Disease Center, Institute for Digestive Research, Soonchunhyang University College of Medicine, Seoul, Republic of Korea; 4 Department of Internal Medicine, Chungbuk National University College of Medicine, Cheongju, Republic of Korea; 5 Division of Gastroenterology and Hepatology, Department of Internal Medicine, Kyungpook National University Hospital, Daegu, Republic of Korea

**Keywords:** Gastroenterology, Medical research

## Abstract

A standardized small bowel (SB) cleansing scale is currently not available. The aim of this study was to develop an automated calculation software for SB cleansing score using deep learning. Consecutively performed capsule endoscopy cases were enrolled from three hospitals. A 5-step scoring system based on mucosal visibility was trained for deep learning in the training set. Performance of the trained software was evaluated in the validation set. Average cleansing score (1.0 to 5.0) by deep learning was compared to clinical grading (A to C) reviewed by clinicians. Cleansing scores decreased as clinical grading worsened (scores of 4.1, 3.5, and 2.9 for grades A, B, and C, respectively, *P* < 0.001). Adequate preparation was achieved for 91.7% of validation cases. The average cleansing score was significantly different between adequate and inadequate group (4.0 vs. 2.9, *P* < 0.001). ROC curve analysis revealed that a cut-off value of cleansing score at 3.25 had an AUC of 0.977. Diagnostic yields for small, hard-to-find lesions were associated with high cleansing scores (4.3 vs. 3.8, *P* < 0.001). We developed a novel scoring software which calculates objective, automated cleansing scores for SB preparation. The cut-off value we suggested provides a standard criterion for adequate bowel preparation as a quality indicator.

## Introduction

Capsule endoscopy (CE) allows direct visualization of the entire small bowel (SB)^1^. It is also safe from sedation-related complications with minimal invasiveness^2^. In current guidelines, CE is the first-line investigation method for patients with obscure gastrointestinal bleeding or suspicious Crohn’s disease^3^. It is also considered as an initial diagnostic modality for various SB diseases including vascular or inflammatory diseases, SB tumors, and polyposis syndrome^3^. With the expansion of CE indications and technological efforts, attempts have been made to observe the entire SB in detail^3–5^. In addition, recently introduced deep learning method has shown excellent performance for detecting SB lesions in CE^6^. It may overcome problems associated with time and effort needed for CE interpretation. Despite these recent advances, CE has a limitation in that its quality is greatly influenced by bowel preparation. In many cases, reading of CE videos is interrupted by air bubbles and residual materials. Inadequate bowel preparation had led to repeat examination and cost increase^7^. As the diagnostic yield of CE highly depends on the preparation quality of passively obtained images, effective bowel cleansing is essential for qualified CE examination. Currently, the guideline recommends bowel preparation quality to be included in the CE report, and the rate of adequate bowel preparation is considered one performance measure^3^. Accordingly, quality control of CE requires an objective scoring system to assess SB preparation. However, a standardized and validated cleansing scale is currently unavailable, which is why the rate of bowel preparation is limited to only minor performance measures.

Several grading scales to assess SB preparation quality have been reported^7–9^. Because the evaluation of bowel preparation using these scales also depends on clinicians’ subjective judgment, validation does not guarantee these scales’ objectivity. Computed cleansing scores using color intensities of tissue color bar (PillCam) or map view (MiroCam) have also been developed^10,11^. They can be integrated into their own CE reading programs to provide objectively calculated scores. However, color intensities of condensed bands are insufficient to fully represent the cleanness of the entire CE image over tens of thousands. Also, if only the intensity of certain colors is recognized, it can be difficult to distinguish between a color due to bleeding or ulcer and a color due to residual materials. Besides, these integrated scales cannot be applied to other CE devices in general.

Thus, the aim of this study was to develop an automated calculation software for SB cleansing score that could represent overall cleanness of the entire CE image to be actually read. This trial is expected to provide an objective cleansing scale for CE and suggest a standard criterion for adequate bowel preparation as a quality indicator.

## Methods

### Study design

**Figure 1:**
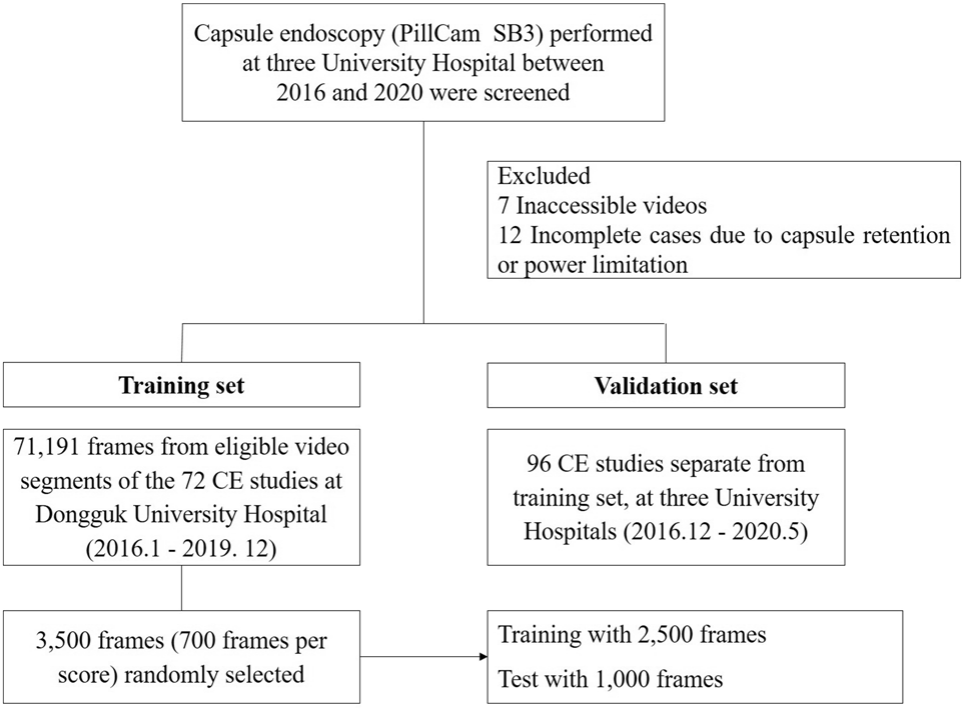
Data flow and deep learning process.

Small bowel CE (PillCam SB3, GIVEN Imaging Ltd., Yoqneam, Israel) cases consecutively performed at three University Hospital (Dongguk Univ., Chungbuk national Univ., and Kyungpook national Univ.) of South Korea between 2016 and 2020 were screened. The SB3 cases with patients over 18 years of age were enrolled. Reasons for CE examinations were obscure gastrointestinal bleeding, suspected or established Crohn’s disease, and suspected small bowel tumor or polyposis. Overnight fasting was performed for all patients. Bowel cleansing was achieved with 2 L polyethylene glycol (PEG) plus ascorbic acid (Coolprep; Taejoon Pharm. Co., Seoul, Korea). Exclusion criteria were inaccessible videos due to mechanical error and incomplete cases when the cecum was not reached due to capsule retention or power limitation. Among eligible CE cases, 72 cases from Dongguk Univ. Hospital between Jan. 2016 and Dec. 2019 were selected for deep learning database (training set). In addition, a separate set of 96 CE cases from three Univ. Hospitals between December 2016 and May 2020 were selected for external validation (validation set) (Fig. 1). The study was conducted in accordance with the guidelines of the Declaration of Helsinki and was approved by the Institutional Review Board of Dongguk University Ilsan Hospital (IRB no. DUIH 2020-06-017). Because this is a retrospective study using CE images that has already completed, informed consent was waived from IRB.

### Data collection for training

**Figure 2:**
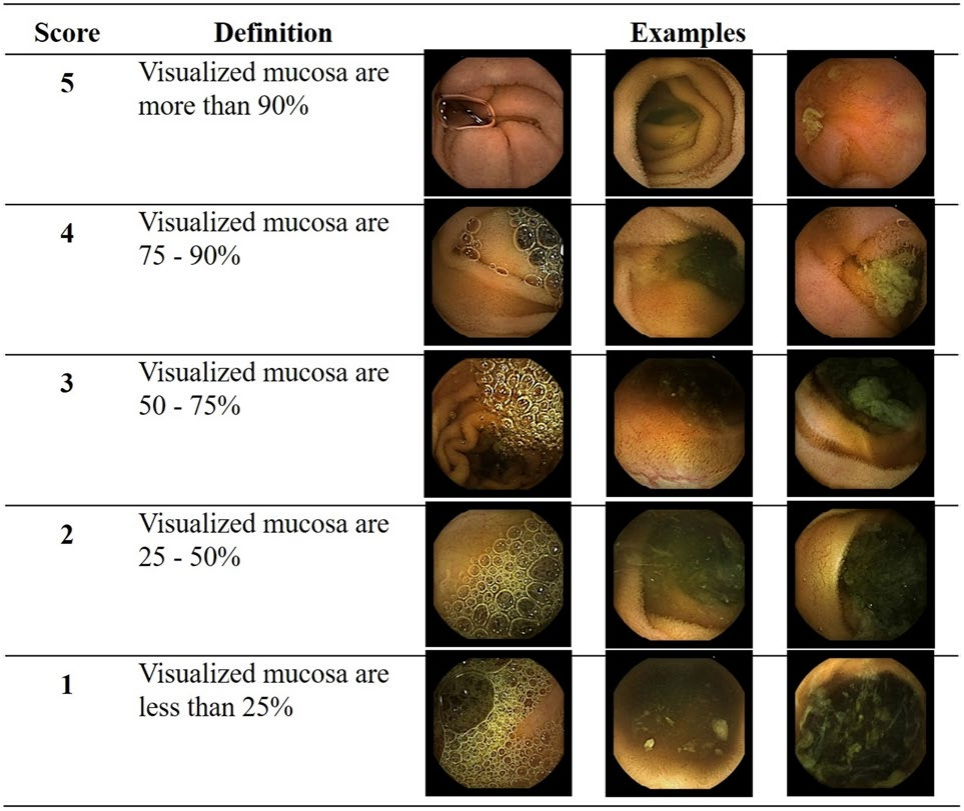
Cleansing score used for deep learning: a 5-step scoring method based on the proportion of visualized mucosa.

As significant abnormalities such as bleeding and ulcer could confuse training of bowel cleansing state, video segments without any significant SB lesion were extracted from cases (n = 72) of the training set (Fig. 1). In sequence, 71,191 frames (still-cut images) were separated from extracted video segments using an OCR (optical character recognition) program. These separated frames were classified into four categories: normal-clean mucosa, bubble-dominant mucosa, bile-dominant mucosa, and debris-dominant mucosa. Two experienced CE readers (J.H.N. and D.J.O.) reviewed these frames and scored cleansing qualities based on the proportion of visualized mucosa (Fig. 2). This scale used 5-step scores ranging from 5 (more than 90% of mucosa visible) to 1 (less than 25% of mucosa visible) depending on obscuration by bubble, bile, and debris. If there were any discrepancies between the two readers, a final score was determined after re-evaluation and discussion with a senior reader (Y.J.L.).

### Deep learning process

To escape from data imbalance problem, 700 images per each cleansing score were selected from the 5-step scored frames to develop a deep learning model that could classify SB cleansing state. A total of 3500 images were randomly separated into 2500 and 1000 images for training and verification, respectively. A deep learning network called InceptionResnetV2 was used for training due to its recent good performance in ImageNet Challenges^12^. Our dataset was not enough to train the deep network from scratch (empty parameter). Therefore, training was started from a pre-trained model parameter using ImageNet dataset. First, the last layer from the pre-trained parameter was trained with hyperparameters of 10,000 for the number of steps, 24 for batch size, and 0.01 for learning rate. Top-1 and Top-2 accuracies of the trained network for the test were 50.4% and 74.5%, respectively. Full layers were then trained with hyperparameters of 220,000 for number of steps, 24 for batch size, and 0.0001 for learning rate. Final Top-1 and Top-2 accuracies of the trained network were 69.4% and 91.2%, respectively. Because the dataset was classified by clinicians subjectively, uncertainty between two scores was allowed. Before a hard determination of the score, the probability for each score was predicted applying softmax function. From the output of soft function, the final cleansing score was estimated by computing expected value as:

$$\mathrm{Final}\_\mathrm{score}\left(\mathrm{I}\right)=\sum_{i=1}^{5}i*{p}_{i}(I)$$

where i indicated the grade and ${\mathrm{p}}_{\mathrm{i}}(I)$ indicated the probability of i-th grade for an image I.

### External validation

The trained scoring software was validated using 96 CE cases different from those used in the training set. All video segments corresponding to SB sections of the validation set were separated into frames using the OCR program. Extracted frames were divided into three equal number of segments according to the time sequence of the video: segment 1 (seg1), proximal third; segment 2 (seg2), middle; and segment 3 (seg3), distal. Using the trained scoring software, a cleansing score was assigned to every frame of the validation set. Separately, two CE readers reviewed bowel preparation quality (clinical grading) of frames. They were blinded to cleansing scores obtained from the trained software, clinical records, and original reports of the validation CE cases. Clinical grading was assessed using a quantitative parameter of a previously validated grading system^13^ based on the proportion of non-prepped images in which bubble, bile, and debris disturbed more than 50% of visualization (Table 1). Clinical grading of each segment (segmental grading, 1 to 4) was assessed independently. Overall image quality (overall grading, A to C) was determined as the sum of segmental grading per CE case. Overall grading of A or B was classified as clinically adequate preparation while overall grading of C was considered as inadequate. Any disagreement between the two readers was resolved after discussion with the senior reader.

**Table 1:** A validated small bowel preparation scale using a quantitative parameter.

**Segmental grading (mucosal invisibility of each segment)**	
Grade 1	< 5% of number of video image^a^ with > 50% invisible mucosa by bubbles, bile, or debris
Grade 2	5–15%
Grade 3	15–25%
Grade 4	> 25%
**Overall grading (overall cleansing quality)**	
Grade A	Total grade 3–5
Grade B	6–8
Grade C	9–12
**Clinically adequate preparation**	
Adequate	Grade A or B
Inadequate	Grade C

^a^Still-cut image (frame).

Capsule endoscopy studies of validation set were prospectively read using an analyzing software (Rapid reader ver. RR83.24.14254.0) for PillCam SB3 by another CE reader (J.P.) who was blinded to results from cleansing scores, clinical grading, and original CE findings. Diagnostic yield was defined as the detection of SB lesion likely to provide diagnostic information such as erosion, ulcer, bleeding, hematin, vascular lesion, and mass.

### Statistical analyses

The main outcome was the performance of deep learning for assessment of SB preparation quality. Average cleansing scores calculated by the deep learning-based software were compared with clinical grading determined using a validated preparation scale.

In the validation set, average cleansing score (from 1.0 to 5.0) per segment and per case were calculated as the sum of cleansing scores divided by the number of frames. ANOVA (analysis of variance) was performed to compare average cleansing scores among different groups of segmental grading (1 to 4) and overall grading (A to C). Post-hoc analysis was performed using Dunnett’s test. Average cleansing scores between clinically adequate and inadequate preparation groups were compared using independent sample *t-*test. Sensitivity and specificity for clinically adequate preparation were calculated for each average cleansing score (1.0 to 5.0). Receiver operating characteristics (ROC) curve was generated to assess a cut-off value of cleansing score for clinically adequate preparation. In addition, whether diagnostic yield differed according to bowel preparation quality was analyzed. Two-sided *P-*values of less than 0.05 were considered statistically significant. All statistical analyses were conducted using SPSS Statistics 19.0 (IBM, Armonk, NY, USA).

## Results

### Descriptive summary and deep learning recognition

Ninety-six CE cases were enrolled for the validation set. Their mean age was 58.1 ± 18.7 years (range, 18–92 years). There were 64 (66.7%) males. Mean SB transit time was 6.1 ± 2.6 h (range, 1.7–13.7 h). Class Activation Map (CAM) applied to the recognition of cleansing score of image frames using the deep learning software was confirmed (Supplementary Figure 1). Lower cleansing score indicated higher weight for bubbles, bile, or debris, whereas higher cleansing score indicated higher weight for clean mucosa and its folds.

### Cleansing scores and clinical grading

The distribution of clinical grading and average cleansing scores per segment is shown in Table 2. There was a tendency for clinical grading to get worse from seg1 to seg3 (*P* = 0.006). More than 60% of seg1 and seg2 had grade 1 whereas only 42.7% of seg3 had grade 1. Grade 4 accounted for 8.3% in seg3. However, it was absent in seg1. Average cleansing scores were also different among segments, showing 4.1 ± 0.5 (range, 2.8–4.9), 4.0 ± 0.7 (range, 1.5–4.9), and 3.7 ± 0.7 (range, 1.4–4.9) for seg1, seg2, and seg3, respectively.

**Table 2:** Clinical grading and cleansing scores of each small bowel segment in the validation set (n = 96).

Grade	Segment 1		Segment 2		Segment 3		*P*-value
	n (%)	Score, mean ± SD	n (%)	Score, mean ± SD	n (%)	Score, mean ± SD	
1	59 (61.5)	4.4 ± 0.3	62 (64.6)	4.3 ± 0.4	41 (42.7)	4.2 ± 0.4	0.006*
2	32 (33.3)	3.8 ± 0.4	26 (27.1)	3.5 ± 0.5	40 (41.7)	4.0 ± 0.5	
3	5 (5.2)	3.3 ± 0.4	2 (2.1)	3.2 ± 0.0	7 (7.3)	3.3 ± 0.5	
4	0 (0)	–	6 (6.3)	2.3 ± 0.5	8 (8.3)	2.2 ± 0.5	
Total		4.1 ± 0.5		4.0 ± 0.7		3.7 ± 0.7	< 0.001*

SD, standard deviation.

*P-values for grade distribution and average cleansing scores per segment, respectively.

**Figure 3:**
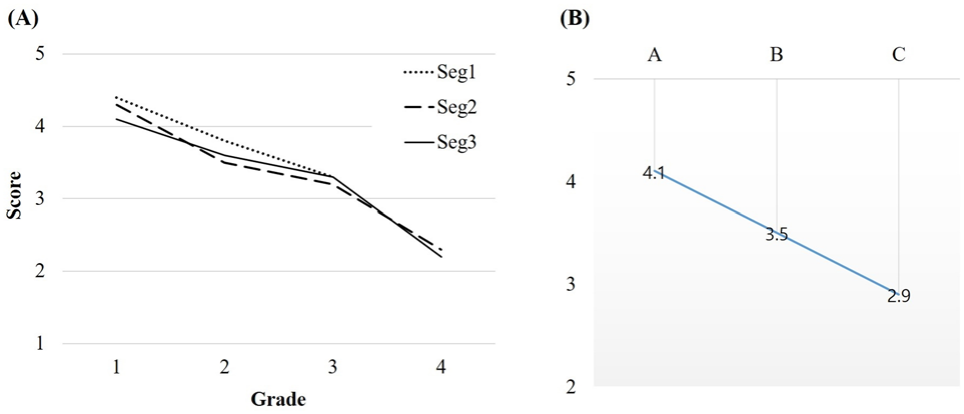
Average cleansing scores by (**A**) segmental grades and (**B**) overall grades. Scores decreased from grade 1 to 4 and from A to C.

Average cleansing scores and segmental grading per segment were analyzed (Table 2 and Fig. 3A). Average cleansing scores tended to decrease from grade 1 to 4 for all segments (all *P* < 0.001). Numbers of cases with overall image quality grades A, B, and C were 75 (78.1%), 13 (13.5%), and 8 (8.3%), respectively. Average cleansing scores decreased when overall grading decreased from grade A to grade C, yielding 4.1 ± 0.4, 3.5 ± 0.5, and 2.9 ± 0.4 for grades A, B, and C, respectively (*P* < 0.001) (Fig. 3B, Supplementary Table 1). Grade A and grade B showed significantly higher average cleansing scores than grade C (*P* < 0.001 and *P* = 0.001 respectively).

**Figure 4:**
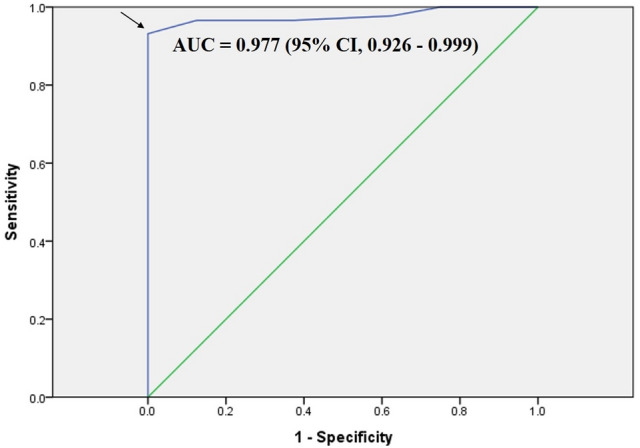
Receiver operating characteristic (ROC) curve of average cleansing score for clinically adequate preparation. The curve estimated a cut-off value of 3.25 (arrow). AUC, area under the curve.

Clinically adequate preparation was achieved for 91.7% (88/96) of cases. The average cleansing score for the adequate group was significantly higher than that for the inadequate group (4.0 vs. 2.9, *P* < 0.001). In ROC curve, a cut-off value of cleansing score at 3.25 for clinically adequate preparation had a sensitivity of 93%, a specificity of 100%, and an AUC (area under the curve) of 0.977 (95% CI: 0.926–0.999, *P* < 0.001) (Fig. 4).

### Diagnostic yield

Main endoscopic findings of the validation set are shown in Table 3. The overall diagnostic yield was 62.5% (60/96). It was not significantly different between adequate and inadequate preparation groups (61.4% vs. 75.0%, *P* = 0.446). The average cleansing score did not differ either according to the overall diagnostic yield (4.0 vs. 3.8, *P* = 0.197). Excluding 36 cases of relatively easy-to-detect lesions such as bleeding, large ulcers, diffuse inflammation or erosions, or mass, detection rate for small lesions such as tiny erosion, aphthous ulcer, hematin, and angioectasia accounted for 41.4% (24/58) in the clinically adequate preparation group. No small lesion was detected in two inadequate cases. Average cleansing score was significantly higher when small lesions were detected (4.3 vs. 3.8, *P* < 0.001). The AUC for cleansing score was 0.747 (95% CI: 0.622–0.871, *P* = 0.001) for detecting the small lesions. The cut-off value of 3.25 of cleansing score for clinically adequate preparation showed 100% sensitivity for diagnosing small lesions.

**Table 3:** Main endoscopic findings of the validation set (n = 96).

CE findings^a^	n (%)
Hematins^b^	1 (1.0)
Erosions^b^	15 (15.6)
Angioectasia^b^	1 (1.0)
Aphthous ulcer^b^	7 (7.3)
Ulcer	18 (18.8)
Bleeding	13 (13.5)
Mass	1 (1.0)
Diffuse inflammation or erosions	4 (4.2)
Total	60 (62.5)

CE, capsule endoscopy.

^a^Classified as a main finding if CE result included various lesions.

^b^These lesions were classified into difficult-to-detect small lesions.

## Discussion

We firstly developed a deep learning-based automation software for calculating SB cleansing score. It is of clinical significance in that it demonstrated a performance of deep learning-based software using a previously validated preparation scale. External validation of this deep learning-based software showed a good performance for preparation quality assessment. In addition, a cut-off value was suggested for clinically adequate preparation.

A recently reported guideline recommends CE indications, cecal visualization, lesion detection, and the rate of adequate bowel preparation as performance measures for qualified CE^3^. Based on 17-year data from the Korean Capsule Endoscopy Registry, one study has shown that inadequate bowel preparation is significantly associated with capsule retention and incomplete examination^14^. Another recent study has shown that higher SB transit time is associated with inadequate bowel preparation^15^. However, the rate of adequate bowel preparation, despite its importance to CE quality, is described as only a minor performance measure in the guideline^3^. The reason is that there is no simplified objective criterion for assessing SB preparation yet. In addition, methods and proper timing of SB preparation remain controversial^16–18^. Unlike colonoscopy, it is not easy to assess the cleanness of tens of thousands of SB images obtained over several hours. No matter how validated scales are used, the current assessment of SB preparation quality by individual clinicians is inevitably subjective and time-consuming. With the expansion of CE indications and recent increase in clinical use, an objective and automated calculation system is essential. The calculating system should be based on clinically validated preparation scales and consistent with experienced CE readers’ assessment. Meanwhile, newly introduced deep leaning-based computational analysis of CE images allows more accurate detection of SB lesions with reduced reading time than conventional CE reading^19–22^. However, as long as the CE subsequently analyze passively obtained images, the performance of deep learning for lesion detection still depends on the quality of bowel preparation. Numerous grading scales with different technical characteristics have been introduced^23^. However, studies on the application of deep learning for assessing SB preparation quality have not been reported yet. Accordingly, authors of this study developed a deep learning-based objective and automated calculating system and showed its clinical usefulness through external validation.

Usually, deep learning-based classification can be applied to explicit problems, for example, object classification of images. Although the dataset in this work was built by CE readers subjectively, the output from the training was significant (Top-2 accuracy of 91.2%). The deep learning model was also validated by comparison with clinical grading. Results of this study demonstrated that deep learning can be applied to subjective problems that are usually determined by human specialists. Our model learned various images of cleanliness for each category of bubble, bile, and debris, but the final cleansing score was derived regardless of the category. Since the duration or degree of mucosal obscuration can vary by category, an advanced model is needed that can differentiate between categories and assign different cleansing scores.

We used a previously validated cleansing scale^8,13^ for CE readers’ clinical grading. By calculating the percentage of frames with more than 50% not visible, it is considered a more detailed and less subjective method among existing preparation scales^3,23^. The original scale rated both mucosal invisibility and fluid transparency independently. In our study, however, we did not rate the fluid transparency separately as the grading of transparency seemed to be more subjective. Instead, we simply included fluid transparency in the grading of mucosal invisibility. We regarded opaque fluid as ‘invisible’ portion, while images showing transparent fluid were considered ‘visible’. As transparent fluid is enough to detect underlying SB lesions, it is feasible to classify it as visible mucosa.

Compared to the grading scale used by CE readers, scoring for the training set required a more visually simplified scale capable of clearly recognizing the cleanliness of each image frame. The cleansing scale of colonoscopy generally uses a 4-level scale based on the amount of fecal residue and turbid fluid, which is also applied to colon CE^24^. For training of cleansing score, it may be common to use a 4-step score depending on the mucosal visibility. However, score 5 (more than 90% mucosa are visible) was separately classified because we needed to train completely cleaned mucosa. In addition, our 5-step scoring system for deep learning enhanced the average cleansing score. Meanwhile, the deep learning process did not train images containing SB lesions such as bleeding or ulcer. Interestingly, the newly developed software calculated cleansing scores comparable to clinical grading results even for CE cases involving SB lesions in the validation test. As proven by the CAM applied to deep learning recognition, the software accurately recognized residual materials such as bubbles, bile, and debris against clean mucosa and its folds.

The present study showed that cleansing score calculated by deep learning model was highly correlated with clinical grading assessed by clinicians. We suggested a significant cut-off value for clinically adequate preparation (AUC, 0.977). Based on the cut-off value of 3.25, it is possible to evaluate whether the CE was qualified and to determine the need for repeat examination or additional diagnostic approach. There was no difference in bowel preparation quality according to overall diagnostic yield. However, the detection of small, hard-to-find lesions such as a few erosions, aphthous ulcers, and vascular lesions was significantly associated with a high average cleansing score. It is conceivable that bleeding or large ulcers can be easily detected and diagnosed even for inadequate bowel preparation cases. Contrary, small lesions are relatively difficult to be detected in an inadequate preparation state. A cut-off value of 3.25 showed 100% sensitivity for diagnosing small lesions. This suggests that such cut-off value for clinically adequate preparation is sufficient for the detection of small lesions.

Our deep learning model was validated with 100 CE images from 3 hospitals, and the clinical characteristics of each case were not included in the analysis. Although reviewers who determined clinical grades were blinded to the cleansing scores and CE findings of each case, a more independent assessment of bowel cleansing would require more CE cases from more hospitals. In addition, this study is currently in a preliminary stage for developing a deep learning model and validating its performance before it is integrated into the CE reading system and applied in real clinical practice. Further studies using prospectively enrolled CE cases should be warranted to demonstrate the validity and reproducibility of our model with real CE cases in clinical practice.

In conclusion, our novel scoring software provides an objective and automated cleansing score for SB preparation in CE. The suggested cut-off value can be used as a criterion as to whether or not the bowel preparation is appropriate to detect SB lesions in clinical practice. This study is expected to provide a standard for adequate bowel preparation in the quality control of CE. The application of the deep learning model enables evaluation of whether the CE examination was appropriate and its results reliable. Additional advances in the model are expected with more CE case experiences in the future.

## Supplementary Information

Supplementary Figure.

Supplementary Table.
