# Supplementary material for: Development of a deep learning-based software for calculating cleansing score in small bowel capsule endoscopy
Source: Sci Rep. 2021 Feb 24;11:4417. doi: 10.1038/s41598-021-81686-7 (PMC7904767; doi:10.1038/s41598-021-81686-7)

**Title:** Development of a deep learning-based software for calculating cleansing score in small bowel capsule endoscopy

Ji Hyung Nam, M.D., Ph.D.[1†], Youngbae Hwang, Ph.D.[2†], Dong Jun Oh, M.D.[1], Junseok Park, M.D.[3], Ki Bae Kim, M.D., Ph.D.[4], Min Kyu Jung, M.D., Ph.D.[5], and Yun Jeong Lim, M.D., Ph.D.[1*]

[1]Division of Gastroenterology, Department of Internal Medicine, Dongguk University Ilsan Hospital, Dongguk University College of Medicine, Goyang, Republic of Korea

[2]Department of Electronics Engineering, Chungbuk National University, Republic of Korea

[3]Digestive Disease Center, Institute for Digestive Research, Department of Internal Medicine, Soonchunhyang University College of Medicine, Seoul, Republic of Korea

[4]Department of Internal Medicine, Chungbuk National University College of Medicine, Cheongju, Republic of Korea

[5]Division of Gastroenterology and Hepatology, Department of Internal Medicine, Kyungpook National University Hospital, Daegu, Republic of Korea

[*]Correspondence: Yun Jeong Lim

[†]These authors contributed equally to this work.

**Supplementary Figure 1. Class Activation Map for recognition of small bowel cleansing scores by deep learning. Red and yellow colors are weighted parts in the recognition.**

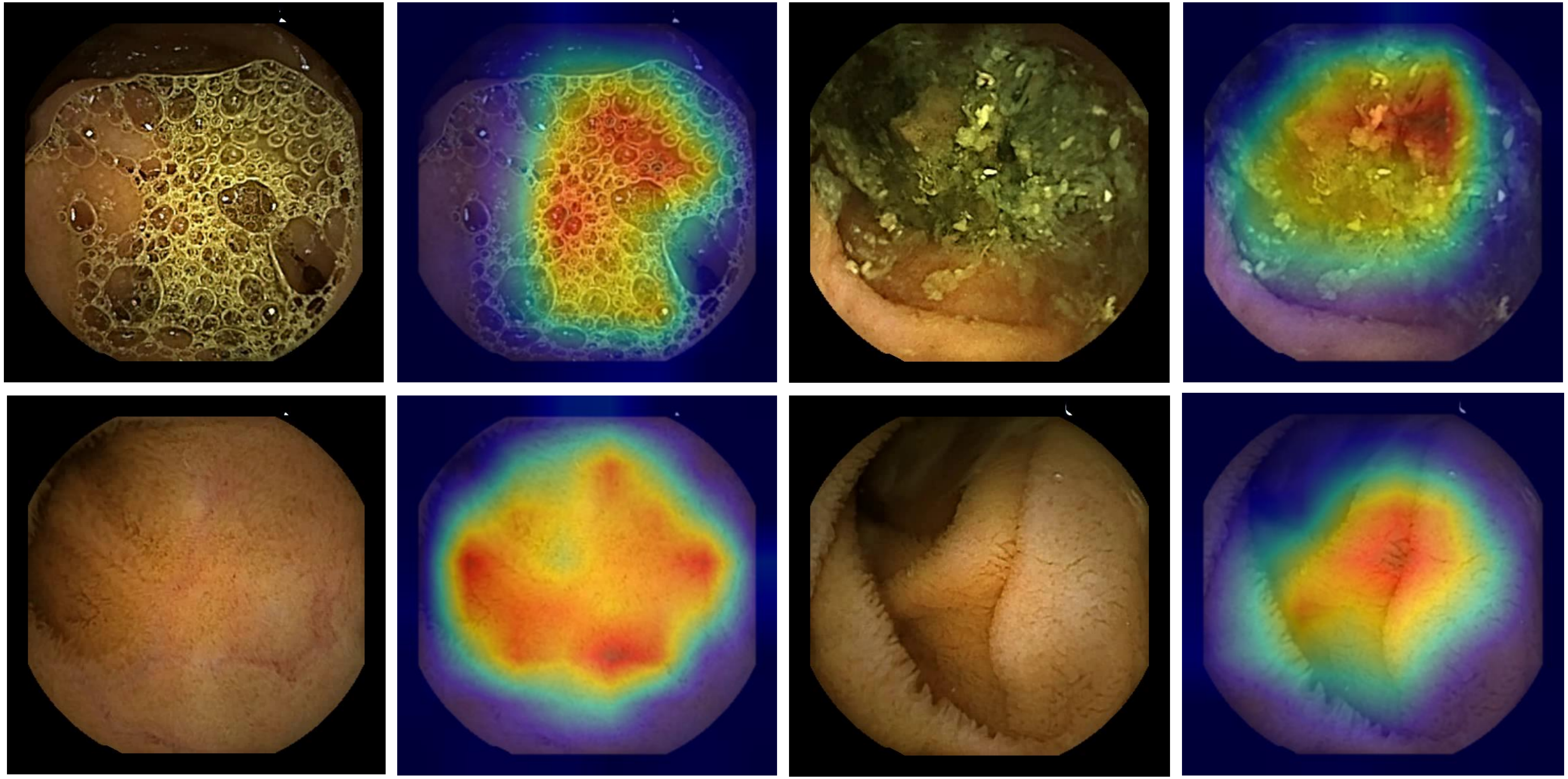

Supplement: Supplementary file 1 — Supplementary Figure. [file 41598_2021_81686_MOESM1_ESM.pdf]
