# Supplementary material for: Development of a deep learning-based software for calculating cleansing score in small bowel capsule endoscopy
Source: Sci Rep. 2021 Feb 24;11:4417. doi: 10.1038/s41598-021-81686-7 (PMC7904767; doi:10.1038/s41598-021-81686-7)
Supplement: Supplementary file 2 — Supplementary Table. [file 41598_2021_81686_MOESM2_ESM.docx]

**Title:** Development of a deep learning-based software for calculating cleansing score in small bowel capsule endoscopy

Ji Hyung Nam, M.D., Ph.D.^1†^, Youngbae Hwang, Ph.D.^2†^, Dong Jun Oh, M.D.^1^, Junseok Park, M.D.^3^, Ki Bae Kim, M.D., Ph.D.^4^, Min Kyu Jung, M.D., Ph.D.^5^, and Yun Jeong Lim, M.D., Ph.D.^1*^

^1^Division of Gastroenterology, Department of Internal Medicine, Dongguk University Ilsan Hospital, Dongguk University College of Medicine, Goyang, Republic of Korea

^2^Department of Electronics Engineering, Chungbuk National University, Republic of Korea

^3^Digestive Disease Center, Institute for Digestive Research, Department of Internal Medicine,

Soonchunhyang University College of Medicine, Seoul, Republic of Korea

^4^Department of Internal Medicine, Chungbuk National University College of Medicine, Cheongju, Republic of Korea

^5^Division of Gastroenterology and Hepatology, Department of Internal Medicine, Kyungpook National University Hospital, Daegu, Republic of Korea

^*^Correspondence: Yun Jeong Lim

^†^These authors contributed equally to this work.

**Supplementary Table 1.** **Comparison of small bowel cleansing scores according to overall grading of cleansing quality**

| Overall grading | No (%) | Score, mean ± SD | 95% CI | *P*-value |
| --- | --- | --- | --- | --- |
| A | 75 (78.1) | 4.1±0.4 | 4.0 – 4.2 | <0.001 |
| B | 13 (13.5) | 3.5±0.5 | 3.2 – 3.8 | 0.001 |
| C | 8 (8.3) | 2.9±0.4 | 2.5 – 3.2 | ref. |

CI, confidence interval; SD, standard deviation.
